# Cadmium and Lead Content in Selected Fungi from Poland and Their Edible Safety Assessment

**DOI:** 10.3390/molecules26237289

**Published:** 2021-11-30

**Authors:** Michalina Gałgowska, Renata Pietrzak-Fiećko

**Affiliations:** 1 Department of Meat Technology and Chemistry, Faculty of Food Sciences, University of Warmia and Mazury in Olsztyn, Cieszyński 1 Sq, 10719 Olsztyn, Poland; michalina.galgowska@uwm.edu.pl; 2 Department of Commodities and Food Analysis, Faculty of Food Sciences, University of Warmia and Mazury in Olsztyn, Cieszyński 1 Sq, 10719 Olsztyn, Poland

**Keywords:** heavy metals, wild mushrooms, environmental pollution, food quality

## Abstract

Mushrooms are able to accumulate toxic trace elements. This study investigates the content of cadmium (Cd) and lead (Pb) in selected species of fungi (*Boletus badius*, *Boletus edulis*, and *Cantharellus cibarius*) from the northeastern part of Poland and estimates their edible safety. The amount of Cd and Pb was determined by flameless atomic spectrometry using the iCE 3000 Series-Thermo. The mean content of Cd in analyzed mushrooms ranged from 0.370 to 2.151 mg/kg d.w., while Pb was found at the level of 0.243–0.424 mg/kg d.w. *Boletus edulis* was characterized by the highest content of Cd, whereas *Cantharellus cibarius* contained the biggest amount of Pb. Estimated exposure to the Cd intake expressed as percentage share in TWI (Tolerable Weekly Intake) was at the highest level in *Boletus edulis* (30.87%), which could be associated with the risk of excessive Cd accumulation in the body.

## 1. Introduction

In terms of natural environment, the northeastern part of Poland is one of the least degraded areas in the country. This part of land covers the so-called “green lungs” territory, which includes the cleanest and naturally unique regions. The area is rich in forests, the properties of which favor the occurrence of undergrowth, e.g., fungi. Some of the most appreciated mushrooms are *Boletus edulis*, *Boletus badius*, and *Cantharellus cibarius* [1].

Fungi are a highly biodiverse group of organisms and they play many roles in nature, the economy, environmental science, food science, and health [2,3]. The collection of wild-growing mushrooms for consumption, recreational, or medicinal purposes dates as far back as from ancient times [4]. Edible fungi are regarded as a delicacy in many countries. They are widely consumed because of their sensory characteristics and attractive culinary attributes. The consumption of mushrooms has continuously increased over time due their high nutritional value, higher than in any vegetable or fruit, and health benefits [5]. Fungi are considered a healthy food, because they are a source of many important nutritional components, including proteins, minerals, carbohydrates, and vitamins. Additionally, the energy they provide is relatively low [6]. They also contain the bioactive compounds, including e.g., phenolics, polyketides, terpenes, terpenoids, steroids, tocopherols, carotenoids, lectins, polysaccharides, ascorbic acid, polyunsaturated fatty acids, ergosterol, being even more abundant than those found in most vegetables and fruits [7,8]. Numerous bioactive compounds are responsible for mushrooms’ antioxidant properties, and fungi seem useful as a natural source of potential antioxidant additives [9,10]. Mushrooms are also regarded as important sources of dietary fiber, consisting mainly of chitin and β-glucans [11]. Furthermore, fungi and their components exhibit health benefits, for instance anti-inflammatory, hepatoprotective, radical scavenging, antiallergic, antimicrobial, and antiviral properties [12,13,14,15]. 

Mushrooms are well known to efficiently absorb and bioaccumulate metalloids and micro- and macroelements due to the specific structure of mycelium, the exposed surface of vegetative cells, and large hyphae surfaces. Edible mushrooms play an important role in the process of elemental cycling and transformation. Their fruiting bodies typically accumulate heavy metals such as As, Cd, Cr, Hg, and Pb. In contrast to vascular plants, fungi are able to accumulate high concentrations of minerals, even when grown on soils with low metal content. This is due to the genetic characteristics of the species, which include the abundance of transport genes and binding ligands [16,17,18,19]. Some minerals are accumulated proportionally in mushrooms (Al, Ba, Fe, Mg, Ni, Ca, Cr); thus, a high concentration of an element in the environment is reflected in its high accumulation in the fruiting body [20]. Other elements, e.g., Cd and Hg, are accumulated disproportionally, and the high content in fungi is caused not by a high abundance of the element in the environment but by the intensive absorption from the environment. Given their constant exposure to pollutants present in their natural habitat, mushrooms have the potential to be good indicators of environmental pollution [21,22,23].

There are various sources of human exposure to toxic elements, including the consumption of contaminated food and water, as well as inhalation of air pollutants or skin contact with them. People who are not at work risk are most susceptible to contamination with heavy metals from the diet. Cd and Pb are undesirable elements for the human body that cause a toxic effect. Cadmium is one of the most important environmental pollutants because it poses a threat to human and animal health. The molecular toxicity of Cd results, inter alia, from interactions between this metal and various ligands containing -OH, -COOH, -NH_2_, -PO_3_H_2_, and imidazole groups in which N and O are electron donors. Such strong interactions can cause changes in molecular conformation, breaking hydrogen bonds or shifting cations away from their sites of action. By reacting with the thiol groups of enzyme proteins, cadmium may affect their activity. Displacing and replacing other metals in metalloenzymes, it can alter the metabolism of proteins, carbohydrates, fatty acids, phospholipids, and nucleic acids. Additionally, by reducing heme concentration, this metal disrupts important intracellular processes. Cadmium creates covalent and ionic bonds with sulfur, oxygen, and hydrogen atoms present in the sulfhydryl, disulfide, carboxyl, imidiazole, or amine groups of many compounds present in cells, causing significant disturbances in their homeostasis. This heavy metal interferes with essential biometals such as zinc, magnesium, selenium, and iron, altering their homeostasis and also disturbing their biological functions. The end result is morphological and functional changes in many organs. Mitochondria are the most vulnerable to the effects of cadmium. Cd^2+^ ions, taking advantage of their similarity to Ca^2+^ ions, penetrate through calcium channels to mitochondrial membranes and modify their permeability. This leads to a disruption of the basic function of the mitochondria: the production of energy for the cell. In addition, changes in the mitochondrial membrane and the resulting disturbances of a number of chemical reactions cause the release of irreversible free radicals that damage these organelles [24,25]. Cd affects proliferation, differentiation, apoptosis, and other cellular activities, and it can cause numerous molecular damage that may be important in carcinogenesis [26]. Lead has been considered the third risky mushroom trace element after cadmium and mercury [13]. The accumulation of Pb in the body leads to the disturbance of the functions of structural proteins and in the metabolic transformations of the cell, such as the regulation of energy processes and the synthesis of proteins and nucleic acids. Lead primarily interferes with heme synthesis in bone marrow erythroblasts by inhibiting δ-aminolevulinic acid dehydratase. Another important mechanism of lead-induced oxidative stress is the effect on the antioxidant defense systems of cells. Lead has a high affinity for sulfhydryl groups and can interfere with antioxidant activity by inhibiting functional SH groups in several enzymes such as catalase, glutathione peroxidase, superoxide dismutase, and glucose-6-phosphate dehydrogenase. In addition, it binds to sulfhydryl proteins, hinders the absorption of trace elements, interrupts the synthesis of structural proteins and calcium, and increases the content of fatty acids in the cell membrane [27].

Information on the chemical composition of food is important when estimating the consumption of essential nutrients but also when assessing the health quality of raw materials and food products. From the toxicological point of view, it is important to study the composition of the content of harmful compounds, including trace metals in food [11]. Therefore, the aim of the study was to determine the content of Cd and Pb in popular species of mushrooms (*Boletus badius*, *Boletus edulis*, *Cantharellus cibarius*) from the northeastern part of Poland and estimate their edible safety.

## 2. Results

**Figure 1:**
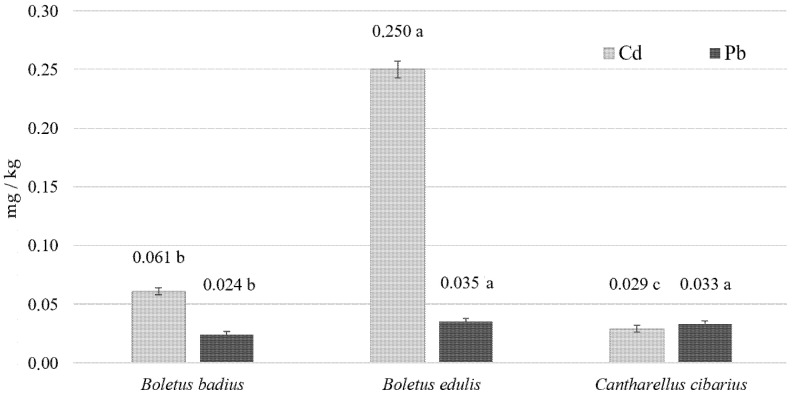
Mean content of Cd and Pb in analyzed mushrooms (mg/kg of wet weight); *n* = 92. Values with the same letters (a, b, c) do not differ significantly among the species at the significance level *p* < 0.05.

The results of the analysis are presented in Table 1 and Table 2 and Figure 1. Throughout the discussion of the results, the mushrooms will be called bay bolete (*Boletus badius*), king bolete (*Boletus edulis*), and chanterelles (*Cantharellus cibarius*). The mean content of Cd in bay bolete, king bolete, and chanterelles ranged from 0.362 to 2.490 mg/kg d.w., while the mean content of Pb ranged from 0.218 to 0.427 mg/kg d.w.

As far as Cd is concerned, statistical differences between regions were observed only in case of king bolete. The contents of Pb in the regions under study did not differ significantly among each species. The highest mean content of Cd was determined in king bolete (2.151 mg/kg d.w.), which differed statistically from values obtained for bay bolete (0.615 mg/kg d.w.) and chanterelles (0.370 mg/kg d.w.). The significantly biggest amount of Pb in analyzed mushrooms was found in chanterelles (0.424 mg/kg d.w.). The results for Pb in king bolete and bay bolete were at a similar level (0.301 and 0.243 mg/kg d.w.) (Table 1).

Mushrooms’ edible safety assessment referred to the Tolerable Weekly Intake (TWI) of Cd and Provisional Tolerable Weekly Intake (PTWI) of Pb, which are presented in Table 2. The TWI for the analyzed mushrooms was calculated according to estimates published on government websites [28], which inform that the average consumption of forest mushrooms in Poland is several kilograms per year per capita, but among people living in large forest areas, it may reach approximately 35 kg per person throughout the year. Due to the fact that in the studied region, there are large open forest areas and the inhabitants obtain mushrooms for individual use, the data on consumption of fungi by local population are not available. Therefore, for the estimation of TWI and PTWI, the obtained results were compared to the average national total mushroom consumption. Based on the above data, the average annual consumption of mushrooms in Poland was estimated at 14 kg of fresh mushrooms; then, a weekly consumption was assumed at the level of approximately 250 g, which in terms of dry weight is approximately 25 g. This value was used to estimate the weekly risk of Cd and Pb uptake with analyzed mushrooms.

**Table 2:** Estimated exposure to the intake of Cd and Pb with the analyzed mushrooms considering assumed weekly consumption at the level of approximately 25 g d.w.

		*Boletus badius*		*Boletus edulis*		*Cantharellus cibarius*	
		Mean Content [mg/25 g d.w.]	Percentage Share in TWI/PTWI	Mean Content [mg/25 g d.w.]	Percentage Share in TWI/PTWI	Mean Content [mg/25 g d.w.]	Percentage Share in TWI/PTWI
Region 1	Cd	0.017	9.71%	0.062	35.43%	0.009	5.14%
	Pb	0.007	0.40%	0.008	0.46%	0.011	0.63%
Region 2	Cd	0.014	8.00%	0.045	25.71%	0.009	5.14%
	Pb	0.005	0.29%	0.007	0.40%	0.011	0.63%
Total	Cd	0.015	8.57% b	0.054	30.87% a	0.009	5.14% b
	Pb	0.060	0.34% c	0.008	0.46% b	0.011	0.63% a

Developed on the basis of WHO [29]; Tolerable Weekly Intake (TWI) for Cd = 0.0025 mg/kg of b.w. per week; Provisional Tolerable Weekly Intake (PTWI) for Pb = 0.025 mg/kg of b.w. per week per person with an average weight of 70 kg. Values with the same small letters (a, b, c) do not differ significantly among the species at the significance level *p* < 0.05.

The statistically biggest mean percentage share in TWI for Cd was determined in king bolete (30.87%), while in bay bolete and chanterelles, it was 8.57% and 5.14%. In the case of Pb, the significant differences were observed between all the species; however, the highest share was observed for chanterelles (0.63%). 

## 3. Discussion

The Cd and Pb content varied between species. The content of Cd in king bolete was 3.5 times higher than in bay bolete and almost six times higher than in chanterelles. On the other hand, the highest Pb content was determined in chanterelles. It was 30% higher than that in king bolete and about 40% higher than that of bay bolete. The differences in the content of the analyzed metals between individual species of fungi may result from their different morphological and biological classification and individual bioaccumulation capacity. In the case of regions, in Region 1, slightly higher contents of both Cd and Pb were determined in mushrooms under study. However, these differences were significant only in the case of Cd in king bolete. Among the factors determining the content of the analyzed metals in individual regions, one can mention the diversity of stands, a different composition of forest litter, and a different class of soils. Moreover, regions differed in the degree of urbanization, which is related to the amount of environmental pollutants emitted to soil, water, and air.

Table 3 presents the results of Cd and Pb content in bay bolete, king bolete, and chanterelles from Poland, European countries (Spain, Italy, Slovak Republic, Romania), and others (Africa, China, Turkey) published by various authors.

Due to the diverse environmental and climatic conditions in European countries and these on other continents, the results of the research of the content of heavy metals in mushrooms have different values. The Cd content obtained in this study in bay bolete was comparable to the results presented by Adamiak et al. [30]. On the other hand, much higher amounts of Cd were determined by Karmańska and Wędzisz [31] and Mleczek et al. [32]. From 46 to 100% higher cadmium Cd content in bay bolete was found in Łódzkie Voivodship [32]. Mleczek et al. [32] presented values from 1.47 to 1.89 mg/kg d.w., which are 140–207% higher compared to the results obtained as part of this work. In the case of Pb, its similar contents in bay bolete were determined by Adamiak et al. [30], while Mleczek et al. [32] obtained results from two to almost five times higher. In conclusion, the results concerning bay bolete obtained in this work were not as high as those determined by other researchers from Poland.

The results of the research on Cd content in Polish king bolete differ depending on the regions of their occurrence. The Cd content in this species was higher than that presented by Adamiak et al. [30], who indicated values from 1.770 to 1.900 mg/kg d.w. However, after unifying the results of Karmańska and Wędzisz [31], the Cd content determined by the authors in king bolete was higher and ranged from 2.450 to 5.600 mg/kg d.w. When examining European mushrooms, Giannaccini et al. [33] obtained values comparable to presented in this work of Cd contents in king bolete. However, Chiocchetti et al. [11], Zavastin et al. [34], and Coroian et al. [35] determined smaller amounts (0.756–1.310, 1.070, and 0.120–1.320 mg/kg d.w.). The highest Cd content in king bolete was found in Yunnan Provinces in China: 5.700–88.450 mg/kg d.w. [36]. The values obtained there were several hundred times higher than those determined in this study. According to Liu et al. [37], Cd contents in mushrooms from a similar area in China ranged from 0.000 to 2.800 mg/kg d.w. The Cd content in the African king bolete was 0.960–1.850 mg/kg d.w. [3]. In the case of Pb in Polish king bolete, Adamiak et al. [30] showed 46–83% higher results. The results most similar to those obtained in this study were presented by Coroian et al. [35] (0.150–0.340 mg/kg d.w.). Other researchers of European mushrooms of this species determined higher Pb content results: 0.800–2.600 mg/kg d.w. [33], 0.626 mg/kg d.w. [34], and 0.094–1.940 mg/kg d.w. [8]. In the studies conducted by Liu et al. [37] on king bolete, 1.300–5.500 mg/kg d.w. of Pb was found, while in South Africa, it was determined from 0.000 to 2.020 mg/kg d.w. Compared to other countries, the studied king bolete was characterized by a relatively high content of both Cd and Pb. Only in China were the values many times greater.

In the case of chanterelles collected in Poland, the results of Cd content closest to those in this study were presented by Falandysz et al. [38] (0.230–1.600 mg/kg d.w.). The lowest content was determined by Mleczek et al. [39] (0.210 mg/kg d.w.) examining mushrooms collected near the heavily trafficked road (0.210 mg/kg d.w.). Karmańska and Wędzisz [31], on the other hand, determined the Cd level to be over five times higher than presented in this study. European chanterelles have various Cd amounts. The lowest content of this element was determined by Zavastin et al. [34] (0.080 mg/kg d.w.). Arvay et al. [40] and Coroian et al. [35] indicated the following values: 0.200–5.700 and 0.600–1.130 mg/kg d.w., respectively, which are higher than those obtained in this work. The most comparable contents were determined by Chiocchetti et al. [11], who had examined other *Cantharellus* species (*Cantharellus lutescens*, *Cantharellus tubaeformis*).

Falandysz et al. [38], examining chanterelles from China, obtained the value of 0.580 mg/kg d.w., while Türkmen and Budur [41] determined 0.200–0.800 mg/kg d.w. of Cd in this species from Turkey. The obtained Pb content in chanterelles is in line with the results presented by Falandysz et al. [38] (0.170–0.660 mg/kg d.w.). Mleczek et al. [39] (0.300 mg/kg d.w.) obtained a lower content than that in this study. In addition, smaller amounts of Pb in chanterelles were determined by Coroian et al. [35] (0.120–0.300 mg/kg d.w.) and Zavastin et al. [34] (0.280 mg/kg d.w.). A wider range of results was presented by Arvay et al. [40] examining mushrooms from the Slovak Republic (0.100–3.200 mg/kg d.w.). The range from 0.219 to 0.645 mg/kg d.w. in mushrooms of the *Cantharellus* family was determined by Chiocchetti et al. [11] from Spain. In China, the Pb content in chantarelles was higher than that in the presented study. Falandysz et al. [38] determined 1.100 mg/kg d.w. in mushrooms from China, while in Turkey, Türkmen and Budur [41] determined 0.340–0.910 mg/kg d.w.

Among all the mushroom species studied, king bolete had the highest Cd content compared to other countries (except China), which suggests the need for further extended research, including the analysis of environmental factors (soil, water, and air contamination) that can determine the presence of metals in fungi.

Comparing the obtained results to the TWI value for Cd, it was found that in the case of king bolete, the percentage share was statistically the biggest and reached the level of 30.87%. However, the share in the rest of studied species was in the range from 5.14 to 8.57%. PTWI values for Pb in bay bolete, king bolete, and chanterelles differed significantly and amounted to 0.34, 0.46, and 0.63%, respectively (Table 2).

Figure 1 shows the results of the average content of Cd and Pb in analyzed species of mushrooms expressed in mg/kg w.w. The calculation was made taking into account the water content in bay bolete, king bolete, and chanterelles, which amounted to 90.1, 88.4, and 92.3%, respectively. The purpose of the calculation was to compare obtained results to maximum levels of Cd and Pb in foodstuffs regulated by Commission Regulations, which are expressed in mg/kg of wet weight.

Table 4 and Table 5 show the maximum levels of Cd and Pb in different foodstuffs. In the Cd regulations (Table 4), the cultivated mushrooms are grouped with leaf vegetables, fresh herbs, leafy brassica, celery, celeriac, parsnips, salsify, horseradish, and the following fungi: *Agaricus bisporus*, *Pleurotus ostreatus*, and *Lentinula edodes*. Other fungi were classified into a separate group for which a five-fold greater tolerance for Cd was established. In the case of Pb (Table 5), fungi are located in two different groups: together with (1) vegetables excluding leafy brassica, salsify, leaf vegetables and fresh herbs, fungi, seaweed, and fruiting vegetables, and (2) leafy brassica, salsify, leaf vegetables excluding fresh herbs, and the following fungi: *Agaricus bisporus*, *Pleurotus ostreatus*, and *Lentinula edodes*. The Pb level values for both these groups are different. It may result from the amount and frequency of consumption of the mentioned raw materials.

Relating the obtained results (Figure 1) to Commission Regulation (EU) No 488/2014 of 12 May 2014 as regards maximum levels of cadmium in foodstuffs [42], it can be observed that none of the mushrooms under study exceeded maximum levels (1 mg/kg w.w) for fungi, excluding *Agaricus bisporus* (common mushroom), *Pleurotus ostreatus* (Oyster mushroom), and *Lentinula edodes* (Shiitake mushroom) (Table 4). 

The Pb results obtained for the mushrooms under study did not exceed the admissible levels up to 0.100 mg/kg w.w. according to Commission Regulation (EU) 2015/1005 of 25 June 2015 as regards maximum levels of lead in certain foodstuffs [43] (Table 5).

## 4. Materials and Methods

### 4.1. Research Material

**Figure 2:**
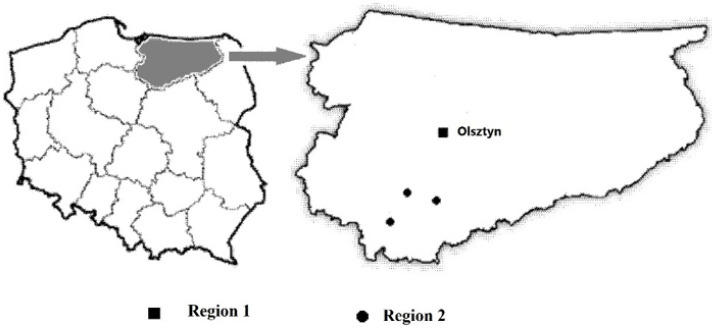
The areas of Warmia and Mazury region, where the samples were collected.

The subject of research in this study was dried edible mushrooms—*Boletus badius* (king bolete), *Boletus edulis* (bay bolete), and *Cantharellus cibarius* (chanterelles)—collected from June to August 2019. The research mushrooms were purchased at local "undergrowth purchase points" in Olsztyn and other regions of Warmia and Mazury, where the residents sell mushrooms harvested in the surrounding forests. Mushrooms at the purchase points were assessed by mushroom experts and sold fresh or processed (including dried) to individual consumption or for further marketing. All samples of the analyzed fungi species were verified in terms of the place of their collection. Mushrooms were divided into lots, one of which came from the vicinity of Olsztyn (Region 1), and another from the three areas 50–100 km away from Olsztyn (Region 2). Region 1 is a city of approximately 170,000 inhabitants with a large industrial enterprise, while Region 2 comprises agricultural land (Figure 2). In total, 92 samples of research mushrooms were collected: approximately 30 of each species. Each single sample was analyzed in triplicate.

### 4.2. Analytical Methods

#### 4.2.1. Samples Preparation

Individual samples of each mushroom species were shredded with a laboratory grinding mill to particles having a diameter of about 0.5 mm. The samples of analyzed mushroom species prepared in this way were weighed into glass test tubes in the amount of about 1 g and subjected to further procedures in order to determine the content of Cd and Pb.

#### 4.2.2. Samples Mineralization

The mineralization of the samples was performed according to the method described by Whiteside and Miner [44]. Weighed samples were mineralized using the "wet" method in a mixture of nitric and perchloric acids (3:1, *v*/*v*) and reagents of purity grade for inorganic trace analysis (Suprapur–Merck, Darmstadt, Germany). The analysis was carried out in an aluminum electric heating block with temperature programming (VELP DK 20-manufactured by VELP Scientifica, Usmate Velate MB, Italy) for 5–6 h with the temperature gradually increasing from 120 to 200 °C. The obtained colorless mineralizate was quantitatively transferred to 50 cm^3^ volumetric flasks and made up to the mark with deionized water (Merck-Millipore Elix Advantage 3, Billerica, MA, USA). Reagent samples were prepared in parallel with the test samples.

#### 4.2.3. Cd and Pb Determination 

The content of Cd and Pb in the mineralized mushroom samples under study was determined by flameless atomic spectrometry using the electrothermal atomizer (GFAAS graphite furnace) in iCE 3000 SERIES-THERMO-spectrometer (Thermo Scientific, Loughborough, England), equipped with a GLITE data station (Zeeman), one-element hollow cathode lamps (HCL) (Thermo Scientific, Loughborough, England), and background correction. Before measuring the cadmium and lead content, the method was optimized. After the modification of the method, the furnace work program consisted of 4 stages, such as: drying, incineration, atomization, burning, and cleaning the litter box. To determine the Cd content, the following temperatures were used at individual stages: 100 °C, 600 °C, 1000 °C, and 2500 °C. The holding times of the individual temperatures were as follows: 30 sec, 20 sec, 3 sec, and 3 sec, respectively. In the case of lead, the following temperatures were used: 300 °C, 600 °C, 900 °C, and 1000 °C. The holding times of the individual temperatures were as follows: 30 sec, 20 sec, 3 sec, and 3 sec, respectively. During the determination of the tested heavy metals, the modifier magnesium nitrate was used at a concentration of 20 µL/mL. Measurements were made at wavelengths: 228.8 nm for Cd and 217.0 nm for Pb.

The selected concentrations of standard solutions of Cd and Pb constituted the measuring range of the analytical method used in the experiment. In order to determine the equation of the relationship between the measurement of the signal generated by the device and the content of the analyte in the sample, calibration curves for individual elements were prepared. To this end, three parallel absorbance measurements were made for each standard solution, starting with the blank measurements. Calibration curves for Cd and Pb were generated from the mean absorbance values. Equations of straight lines describing the curves were determined by the least squares method (linear regression) according to the formula: y = ax + b, where a is the slope (directional) coefficient of the straight line, and b is the straight line shift is the coefficient (Table 6). Based on the regression coefficient (R^2^), the linearity was determined, i.e., the range of the analyte content for which the output signal of the measuring device is proportional to this content. The value of this parameter should meet the condition R^2^ ≥ 0.999. The accuracy of the method was checked on the basis of testing the certified reference material of INCT-TL-1 tea leaves, which were analyzed five times. 

### 4.3. Statistical Methods

Statistical analysis of the results concerning Cd and Pb content in the studied mushrooms was performed using the computer package of Statistica 13.1 (StatSoft Inc., Tulsa, OK, USA) software. The MS Excel spreadsheet was used to present the results. At the beginning, the measures of descriptive statistics such as arithmetic mean, median, variance, standard deviation, and the range were calculated. Afterwards, to check the consistency of the distribution of the examined feature with the normal distribution, the Kolmogorov–Smirnov test was performed. Then, the median series test was carried out to check whether the values had a random distribution. To compare the mean values of features between the samples, non-parametric tests were used. In order to test the differences between several independent samples, the Kruskal–Wallis test was used to verify the null hypothesis assuming "equality of means in the tested samples" with the alternative hypothesis that these means are not equal. Using both tests, the significance level was 0.05. The assumed grouping factor was the species of fungi.

## 5. Conclusions

Health risk related to exposure to heavy metals increases significantly in the case of fungi growing in contaminated and heavily polluted areas, such as the surroundings of mines and metal smelters. Our study aimed to determine the content of Cd and Pb in popular raw material: mushrooms derived from clean and low industrialized areas of Poland. Despite the fact that the Warmia and Mazury region is regarded as the “green lungs” of the country, the contaminants are determined in many raw food materials. In each of the studied species of fungi, both Cd and Pb were found. The obtained results did not exceed the permissible levels of this heavy metals in mushrooms, referring them to the European Union regulations; however, in the case of king bolete, the values were high. This hypothesis is derived from the exposure to the intake of Cd and Pb with the analyzed mushrooms calculation related to TWI and PTWI values. Increased consumption of this mushroom could be associated with the risk of excessive Cd accumulation in the body. This fact may determine the health quality of the studied mushroom, which may pose a health and life risk for humans. The specificity of mushrooms among species and their bioindication abilities may constitute important criteria for determining their health quality and the degree of environmental pollution from which they are collected. A high concentration of Cd in king bolete may indicate that the environment is contaminated with this metal. On the other hand, some fungi are more prone to absorbing and accumulating metals. The presence of high concentrations in these fungi may not reflect the degree of environmental pollution.

## Figures and Tables

**Table 1:** Mean content (±standard deviation) of Cd and Pb in analyzed mushrooms (mg/kg d.w.); *n* = 92.

	*Boletus badius*		*Boletus edulis*		*Cantharellus cibarius*	
	Cd	Pb	Cd	Pb	Cd	Pb
Region 1	0.669 ± 0.022 a	0.268 ± 0.014 a	2.490 ± 0.078 a	0.334 ± 0.019 a	0.377 ± 0.022 a	0.421 ± 0.019 a
Region 2	0.562 ± 0.018 a	0.218 ± 0.011 a	1.811 ± 0.053 b	0.267 ± 0.014 a	0.362 ± 0.025 a	0.427 ± 0.022 a
Total	0.615 ± 0.020 B	0.243 ± 0.013 B	2.151 ± 0.066 A	0.301 ± 0.017 B	0.370 ± 0.024 B	0.424 ± 0.021 A

Values with the same small letters (a, b) do not differ significantly among the species between the regions at the significance level *p* < 0.05; values with the same big letters (A, B) do not differ significantly between the species at the significance level *p* < 0.05, d.w., dry weight.

**Table 3:** Cd and Pb content in *Boletus badius*, *Boletus edulis*, and *Cantharellus cibarius* according to other authors’ studies.

Localization		*Boletus badius*		*Boletus edulis*		*Cantharellus cibarius*		References
		Cd	Pb	Cd	Pb	Cd	Pb	
Poland	Wysoczyzna Siedlecka,	0.721– 0.767	0.142– 0.357	1.770– 1.900	0.440– 0.552	nd	nd	Adamiak et al., 2013
	Poland	nd	nd	nd	nd	0.230– 1.600	0.170– 0.660	Falandysz et al., 2017
	Łódzkie Voivodship,	0.900–1.600	nd	2.450–5.600	nd	1.900– 2.100	nd	Karmańska and Wędzisz, 2010
	Pomeranian, the Greater Poland, the Łódź, the Opole and Silesian region	1.470– 1.890	0.510– 1.100	nd	nd	nd	nd	Mleczek et al., 2013
	Surroundings of a heavily trafficked road	nd	nd	nd	nd	0.210	0.300	Mleczek et al., 2016
Europe	Slovak Republic, the central Spiš region,	nd	nd	nd	nd	0.200– 5.700	0.100– 3.200	Arvay et al., 2014
	Spain	nd	nd	0.756– 1.310	0.094– 1.940	0.194–0.622 *	0.219–0.645 *	Chiocchetti et al., 2020
	Romania, Transylvania	nd	nd	0.120– 1.320	0.150– 0.340	0.600– 1.130	0.120– 0.300	Coroian et al., 2018
	Italy, Lucca province, North-Central	nd	nd	2.000– 3.400	0.800– 2.600	nd	nd	Giannaccini et al., 2012
	Romania, Poiana Stampei area, Suceava County	nd	nd	1.070	0.626	0.080	0.280	Zavastin et al., 2018
Others	China	nd	nd	nd	nd	0.580	1.100	Falandysz et al., 2017
	China Southwest, Yunnan and Sichuan Provinces	nd	nd	0.000– 2.800	1.300– 5.500	nd	nd	Liu et al., 2016
	KwaZulu, South Africa	nd	nd	0.960– 1.850	0.00– 2.020	nd	nd	Rasalanavho et al., 2020
	China, Yunnan Province,	nd	nd	5.700– 88.450	nd	nd	nd	Su et al., 2018
	Turkey’s Black Sea region	nd	nd	nd	nd	0.200– 0.800	0.340– 0.910	Türkmen and Budur, 2018

nd—no data; * data concerns *Cantharellus* family.

**Table 4:** Maximum levels of Cd in foodstuffs.

	Product	The Highest Level of Cd mg/kg of Wet Weight
1	Vegetables and fruit, excluding root and tuber vegetables, leaf vegetables, fresh herbs, leafy brassica, stem vegetables, fungi, and seaweed	0.050
2	Root and tuber vegetables (excluding celeriac, parsnips, salsify, and horseradish), stem vegetables (excluding celery). For potatoes, the maximum level applies to peeled potatoes	0.100
3	Leaf vegetables, fresh herbs, leafy brassica, celery, celeriac, parsnips, salsify, horseradish, and the following fungi: *Agaricus bisporus* (common mushroom), *Pleurotus ostreatus* (Oyster mushroom), and *Lentinula edodes* (Shiitake mushroom)	0.200
4	Fungi, excluding those listed above	1.000
5	Cereal grains excluding wheat and rice	0.100
6	Wheat grains, rice grains, wheat bran, and wheat germ for direct consumption, soy beans	0.200
7	Meat of bovine animals, sheep, pig, and poultry	0.050
8	Muscle meat of fish excluding species: mackerel, tuna, bichique, bullet tuna, anchovy, swordfish, and sardine	0.050
9	Infant formulae and follow on-formulae: - Powdered formulae manufactured from cows’ milk proteins or protein hydrolysates; - Liquid formulae manufactured from cows’ milk proteins or protein hydrolysates powdered formulae manufactured from soya; - Protein isolates, alone or in a mixture with cows’ milk proteins; - Liquid formulae manufactured from soya protein isolates, alone or in a mixture with cows’ milk proteins.	0.010 as from 01/01/2015 0.005 as from 01/01/2015 0.020 as from 01/01/2015 0.010 as from 01/01/2015
10	Food supplements excluding food supplements consisting exclusively or mainly of dried seaweed, products derived from seaweed, or dried bivalve molluscks.	1.000

Developed on the basis of Commission Regulation (EU) No 488/2014 of 12 May 2014 amending Regulation (EC) No 1881/2006 as regards maximum levels of cadmium in foodstuffs (text with EEA relevance).

**Table 5:** Maximum levels of Pb in foodstuffs.

	Product	The Highest Level of Pb mg/kg of Wet Weight
1	Raw milk, heat-treated milk, and milk for the manufacture of milk-based products	0.020
2	Infant formulae and follow-on formulae: - Marketed as powder; - Marketed as liquid.	0.050 0.010
3	Meat of bovine animals, sheep, pig, and poultry	0.100
4	Muscle meat of fish	0.300
5	Cereals and pulses	0.200
6	Vegetables excluding leafy brassica, salsify, leaf vegetables and fresh herbs, fungi, seaweed, and fruiting vegetables	0.100
7	Leafy brassica, salsify, leaf vegetables excluding fresh herbs and the following fungi *Agaricus bisporus* (common mushroom), *Pleurotus ostreatus* (Oyster mushroom), and *Lentinula edodes* (Shiitake mushroom)	0.300
8	Fruit, excluding cranberries, currants, elderberries, and strawberry tree fruit	0.100
9	Fats and oils, including milk fat	0.100
10	Food supplements	3.000

Developed on the basis of Commission Regulation (EU) 2015/1005 of 25 June 2015 amending Regulation (EC) No 1881/2006 as regards maximum levels of lead in certain foodstuffs (Text with EEA relevance).

**Table 6:** Measuring range of the calibration curve, the equation of the calibration curve, and the regression coefficient (R^2^) of Cd and Pb.

Heavy Metal		Calibration Curve Measuring Range (μg/mL)	Calibration Curve Equation	Regression Coefficient (R^2^)
Cadmium	Cd	0.02–0.2	y = 0.3167x + 0.0003	0.9996
Lead	Pb	0.002–0.008	y = 16.446x + 0.0065	0.9997

## Sample Availability

Samples of the compounds are not available from the authors.
